# MFG-E8 regulated by miR-99b-5p protects against osteoarthritis by targeting chondrocyte senescence and macrophage reprogramming via the NF-κB pathway

**DOI:** 10.1038/s41419-021-03800-x

**Published:** 2021-05-25

**Authors:** Yuheng Lu, Liangliang Liu, Jianying Pan, Bingsheng Luo, Hua Zeng, Yan Shao, Hongbo Zhang, Hong Guan, Dong Guo, Chun Zeng, Rongkai Zhang, Xiaochun Bai, Haiyan Zhang, Daozhang Cai

**Affiliations:** 1 Department of Joint Surgery, Center for Orthopaedic Surgery, The Third Affiliated Hospital of Southern Medical University, Guangzhou, China; 2 Department of Orthopedics, Orthopedic Hospital of Guangdong Province, Academy of Orthopedics of Guangdong Province, The Third Affiliated Hospital of Southern Medical University, Guangzhou, China; 3 The Third School of Clinical Medicine, Southern Medical University, Guangzhou, China; 4 Guangdong Provincial Key Laboratory of Bone and Joint Degeneration Diseases, Guangzhou, China

**Keywords:** Predictive markers, Osteoarthritis, Pathogenesis

## Abstract

Milk fat globule-epidermal growth factor (EGF) factor 8 (MFG-E8), as a necessary bridging molecule between apoptotic cells and phagocytic cells, has been widely studied in various organs and diseases, while the effect of MFG-E8 in osteoarthritis (OA) remains unclear. Here, we identified MFG-E8 as a key factor mediating chondrocyte senescence and macrophage polarization and revealed its role in the pathology of OA. We found that MFG-E8 expression was downregulated both locally and systemically as OA advanced in patients with OA and in mice after destabilization of the medial meniscus surgery (DMM) to induce OA. MFG-E8 loss caused striking progressive articular cartilage damage, synovial hyperplasia, and massive osteophyte formation in OA mice, which was relieved by intra-articular administration of recombinant mouse MFG-E8 (rmMFG-E8). Moreover, MFG-E8 restored chondrocyte homeostasis, deferred chondrocyte senescence and reprogrammed macrophages to the M2 subtype to alleviate OA. Further studies showed that MFG-E8 was inhibited by miR-99b-5p, expression of which was significantly upregulated in OA cartilage, leading to exacerbation of experimental OA partially through activation of NF-κB signaling in chondrocytes. Our findings established an essential role of MFG-E8 in chondrocyte senescence and macrophage reprogramming during OA, and identified intra-articular injection of MFG-E8 as a potential therapeutic target for OA prevention and treatment.

## Introduction

Osteoarthritis (OA) is a chronic and highly prevalent joint disease, causing pain and disability in aging population, representing an enormous clinical and financial burden. It begins with destructive changes in the cartilage and progresses to joint space narrowing, synovial inflammation, subchondral bone sclerosis and osteophyte formation, with the manifestations of joint pain, deformity and dysfunction^1^. The Worldwide Report claims that more than 250 million people have suffered from OA up to 2019, which causes tremendous health-care and societal costs by impairing patients’ work productivity and advancing the retirement age^2^. As predicted, by 2040 OA might be the most common form of musculoskeletal disease, which leads to disability^3^.

A large amount of research has been carried out to elucidate the specific mechanism of OA pathogenesis. It is widely accepted that an imbalance of extracellular matrix (ECM) homeostasis triggers cartilage destruction in OA. Integral ECM is essential to the normal load-bearing function of articular cartilage. Moreover, the ECM regulates most cellular behaviors and is necessary for major developmental processes^4^. However, in OA, a number of matrix-degrading enzymes, including matrix metalloproteinases (MMPs), and a disintegrin and metalloproteinases with thrombospondin motifs (ADAMTS) are significantly upregulated, which accelerates the remodeling and loss of flexibility of the ECM even before cartilage destruction occurs^5^. Even worse, ECM damage inevitably leads to abnormal behavior of chondrocytes, which further exacerbates OA progression. Another factor closely correlated with ECM degradation and chondrocyte abnormality is cellular senescence. Senescent chondrocytes exhibit a suppressed regenerative capacity and a loss of original phenotype^6^. Moreover, aging chondrocytes manifest a senescence-associated secretory phenotype (SASP), characterized by enhanced secretion of pro-inflammatory factors such as interleukin-1β (IL-1β), interleukin-6 (IL-6), and tumor necrosis factor α (TNF-α), increased production of ECM degrading enzymes, and accumulated oxidative stress, which exacerbate cartilage damage and promote OA development^7,8^.

Studies also attach great importance to the role of synovitis in OA. Synovial inflammation has been demonstrated to be the precursor of OA onset^9^. It triggers pannus formation and osteoclastogenesis, enhances adherence of synovial tissue to cartilage, and causes the release of inflammatory mediators, perpetuating the processes of cartilage degradation^10^. The aggregation of polarized macrophages in the intimal lining of the synovium is the main morphological feature of synovitis. During OA development, synovial macrophages are activated, accumulated, and polarized into different subtypes, which can be classically categorized as M1- and M2-polarized macrophages^11^. M1 macrophages produce an excess of proinflammatory mediators, including IL-1, IL-6, IL-12, TNF-α, and cyclooxygenase-2 (COX-2). Conversely, M2 macrophages, also known as wound-healing macrophages, manifest an anti-inflammatory function and are conducive to tissue repair and remodeling^11,12^. Using mouse models with enhanced M1 or M2-polarized macrophages, we previously demonstrated that synovial macrophage M1 polarization accelerates experimental collagenase-induced OA progression while M2 polarization significantly alleviates OA development^13^. However, a comprehensive understanding of OA pathogenesis is yet to be achieved.

Milk fat globule-epidermal growth factor (EGF) factor 8 (MFG-E8), also known as lactadherin, is a secreted glycoprotein, which is widely expressed throughout the body^14^. It is most well-known for its vital role of coordinating the engulfment of apoptotic cells, termed phagocytosis, which helps the clearance of dying cells and prevents the release of potentially toxic or immunogenic intracellular materials^15^. Another prominent function of MFG-E8 is its anti-inflammatory effect, which has been well verified in neurodegenerative diseases, diabetes and sepsis^14–16^. Recently, more phenotypes related to MFG-E8 have been discovered. Tests showed that MFG-E8 can reprogram macrophages to the anti-inflammatory M2 phenotype, relieve apoptosis, reverse cellular oxidative stress and modulate the balance between osteoblasts and osteoclasts^17–19^. Moreover, it is reported that MFG-E8 is significantly downregulated in synovial fluid and plasma in OA patients^20^. Nevertheless, the effects of MFG-E8 in OA remain unclear.

In the present study, significant suppression of MFG-E8 was detected in OA conditions. Thus, we hypothesized that MFG-E8 may play an important role in OA development. To validate this hypothesis, we illustrated the protective functions of MFG-E8 in primary murine chondrocytes, the RAW264.7 cell line and in surgically induced OA mice. Downregulation of MFG-E8 exacerbated OA, while supplementary administration of MFG-E8 had a therapeutic effect. Moreover, we found that MFG-E8 modulated NF-κB activation, while upregulating miR-99b-5p in OA significantly restrained MFG-E8 expression. A miR-99b-5p/MFG-E8/NF-κB modulation axis was established in OA models.

## Results

### Loss of MFG-E8-expressing chondrocytes and synoviocytes in patients with OA and in OA mice

**Fig. 1:**
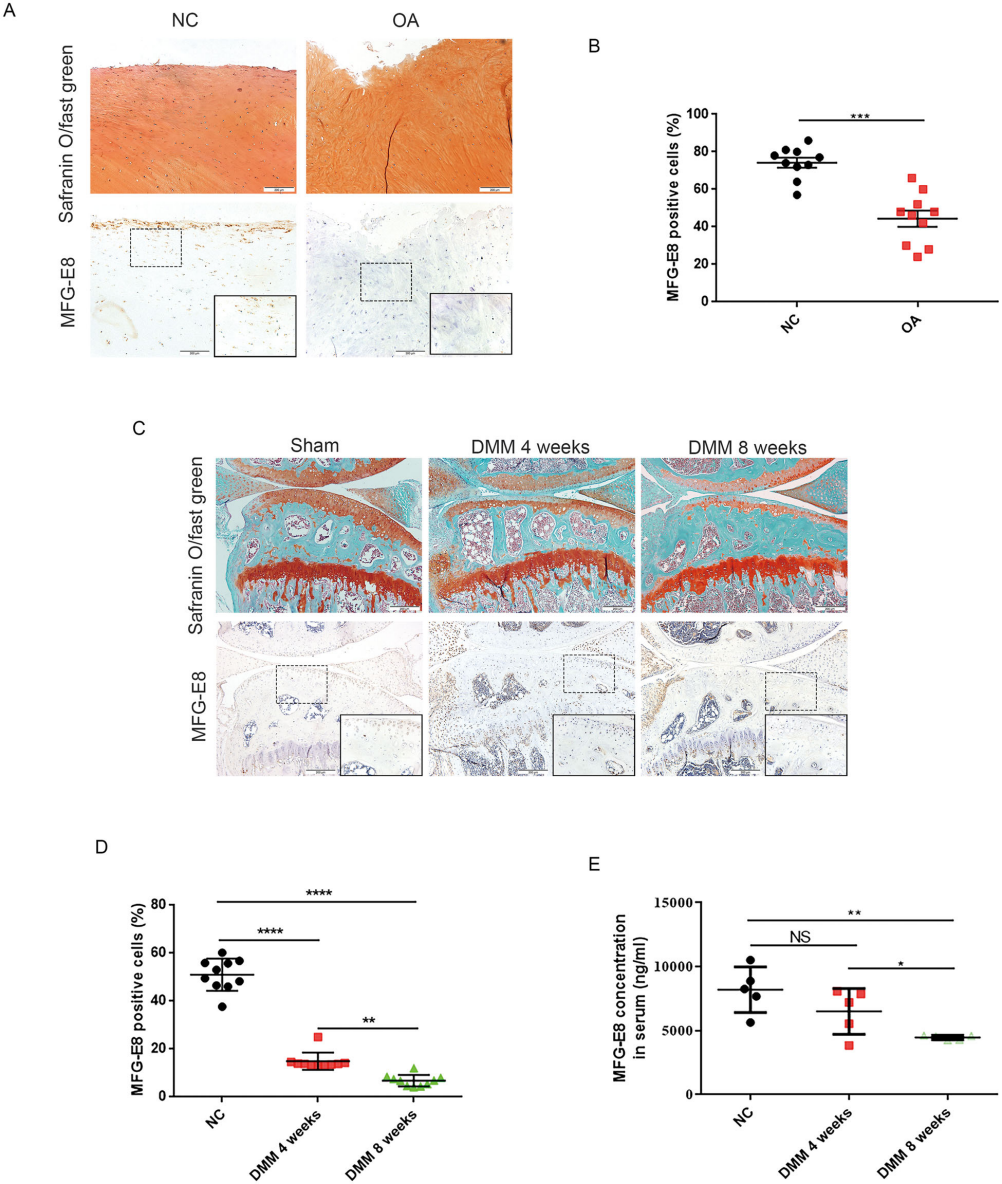
Loss of MFG-E8-expressing chondrocytes and synoviocytes in patients with OA and OA mice. **A** Safranin O and Fast Green staining (upper) and immunostaining of MFG-E8 (lower) of human articular cartilage from medial (OA) and lateral (NC) tibial plateau of OA patients undergoing total knee arthroplasty. Scale bar: 200 µm; **B** Quantification of MFG-E8 in human articular cartilage of medial and lateral tibial plateau. *n* = 10 per group; **C** Safranin O and Fast Green staining (upper) and immunostaining of MFG-E8 (lower) of sagittal sections of knees from controls and OA model mice at 4 weeks and 8 weeks post destabilization of the medial meniscus (DMM) operations. Scale bar: 200 µm; **D** Quantification of MFG-E8 in articular cartilage of sham and DMM mice. *n* = 10 per group; **E** Enzyme-linked immunosorbent assay (ELISA) of MFG-E8 in serum of controls and OA model mice at 4 weeks and 8 weeks post OA surgery. *n* = 5 per group; ^*^P < 0.05, ^**^P < 0.01, ^***^P < 0.001, ^****^P < 0.0001, ns not significant.

To explore whether MFG-E8 plays a role in OA development, we first examined the expression of MFG-E8 in human OA cartilage. Results from immunohistochemistry (IHC) staining revealed that MFG-E8 was decreased in damaged cartilage from the medial tibial plateau of OA patients undergoing total knee arthroplasty compared to cartilage samples from the lateral tibial plateau, which was only slightly damaged (Fig. 1A, B). We then analyzed the expression of MFG-E8 during OA progression using a destabilization of the medial meniscus surgery (DMM)-induced OA mouse model. Similarly, a number of MFG-E8-expressing chondrocytes were observed in normal cartilage, while MFG-E8 showed a progressive downregulation along with increased cartilage damage in OA mice (Fig. 1C, D). Interestingly, enzyme linked immunosorbent assay (ELISA) revealed a significant decrease of MFG-E8 levels in serum from mice with DMM surgery-induced OA (Fig. 1E). Furthermore, in vitro study in IL-1β-treated primary murine chondrocytes confirmed the decreased expression of MFG-E8 during OA (Fig. S1A–C). Besides chondrocytes, we also noticed decreased expression of MFG-E8 in the synoviocytes, especially the macrophage-like synoviocytes, as OA deteriorated in DMM mice (Fig. S1D-E). Moreover, western blot showed that MFG-E8 was sharply downregulated with the increased M1 macrophage polarization in vitro (Fig. S1F). Collectively, these results indicated that MFG-E8 expression was downregulated both locally and systemically as OA progresses, suggesting a potential role of MFG-E8 in OA pathogenesis.

### Loss of MFG-E8 causes striking progressive articular cartilage loss, synovial hyperplasia, and massive osteophyte formation in OA mice

**Fig. 2:**
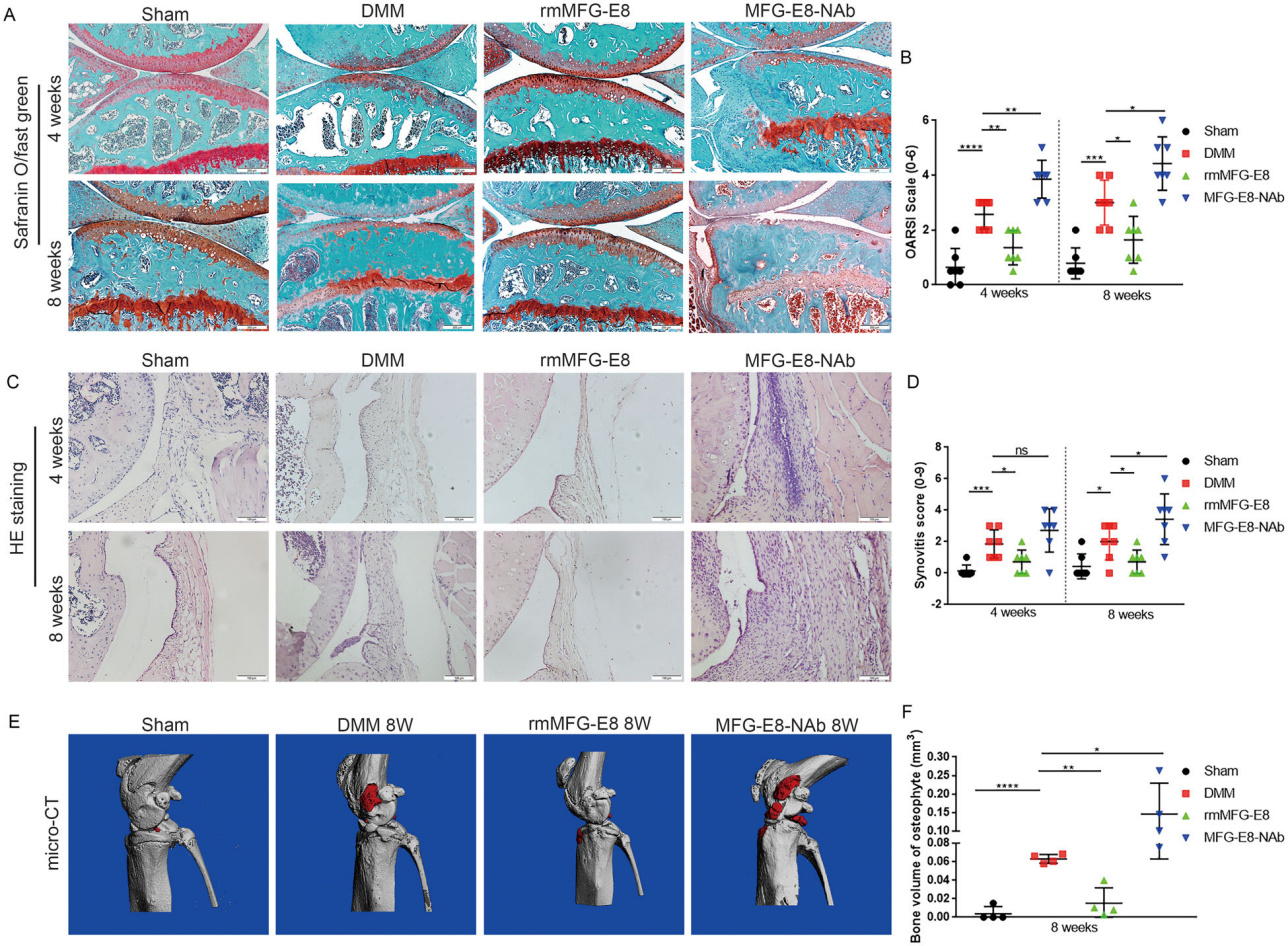
MFG-E8 inhibition causes strikingly progressive articular cartilage loss, synovial hyperplasia, and massive osteophyte formation in OA mice. **A** Safranin O and Fast Green staining of knee cartilage from controls and DMM mice treated with vehicle, recombinant mouse MFG-E8 (rmMFG-E8) and MFG-E8 neutralizing antibody (MFG-E8-NAb) for 4 weeks and 8 weeks. Scale bar: 200 µm; **B** Osteoarthritis Research Society International (OARSI) grades for the joints described in **A**. *n* = 7 per group; **C** H&E staining of synovial tissues from controls and mice treated with vehicle, rmMFG-E8 and MFG-E8-NAb for 4 weeks and 8 weeks. Scale bar: 100 µm; **D** Synovitis score for the joints described in **C**. *n* = 7 per group; **E** Micro-CT scan and three-dimensional reconstruction of the knee joint from controls and mice treated with vehicle, rmMFG-E8, or MFG-E8-NAb for 8 weeks; **F** Bone volume of osteophytes of the knee joints described in **E**. *n* = 4 per group. ^*^P < 0.05, ^**^P < 0.01, ^***^P < 0.001, ^****^P < 0.0001, ns not significant.

To further determine the role of MFG-E8 in OA, 14-week-old male C57 mice were treated by intraarticular injection of MFG-E8 neutralizing antibody or recombinant protein once a week after DMM surgery. Notably, neutralization of MFG-E8 resulted in a significantly higher OA score than controls, characterized by fewer chondrocytes, worse cartilage erosion and loss of proteoglycans at both 4 weeks and 8 weeks post DMM surgery, as confirmed by the OARSI scale and HC/CC ratio (Fig. 2A, B and Fig. S2A–C). Strikingly, compared to controls, high levels of synovial hyperplasia and abundant cell infiltration were observed in the synovial tissue of OA mice treated with MFG-E8 neutralizing antibody, combined with significantly higher synovitis scores at 8 weeks after surgery, while only a slight increase was observed at 4 weeks (Fig. 2C, D). Similarly, a more severe synovitis was found in collagen induced OA (CIOA) mice co-treated with MFG-E8 neutralizing antibody as compared with control mice (Fig. S2 D, E). Furthermore, we used micro-CT to analyze osteophyte formation in knee joints after MFG-E8 interference at 8 weeks post OA surgery. The total volume of osteophytes was markedly increased with reduction of MFG-E8 (Fig. 2E, F). Excitingly, supplementation with exogenous MFG-E8 prevented progression of OA from cartilage damage to synovial hyperplasia and osteophyte formation (Fig. 2A–F and Fig. S2 A–E). These findings suggested that MFG-E8 played an essential role in OA pathology. MFG-E8 neutralization aggravates, while MFG-E8 supplementation reversed the OA phenotypes, in general.

### MFG-E8 restores chondrocyte homeostasis in OA

**Fig. 3:**
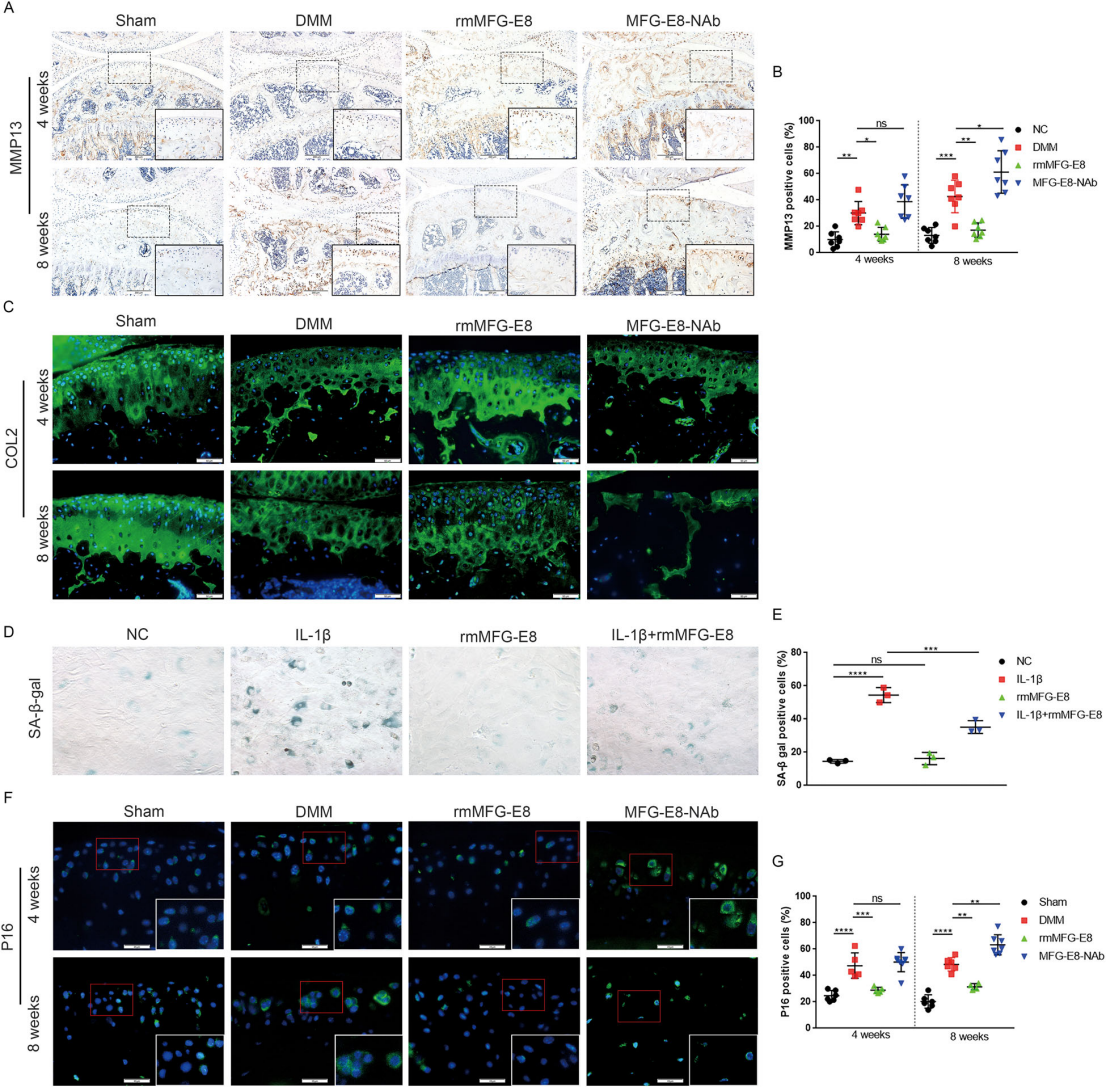
MFG-E8 restored chondrocyte homeostasis and suppressed cellular senescence in OA. **A**, **B** Immunohistochemical staining and quantification of MMP13 in knee cartilage from controls and DMM mice treated with vehicle, rmMFG-E8 and MFG-E8-NAb for 4 weeks or 8 weeks. *n* = 7 per group; Scale bar: 200 µm; **C** Immunofluorescence staining of Collagen II in articular cartilage of sham mice and DMM mice treated with vehicle, rmMFG-E8 or MFG-E8-NAb. *n* = 7 per group; Scale bar: 50 µm; **D**, **E** Senescence-associated β-galactosidase and quantification of IL-1β and rmMFG-E8-treated primary murine chondrocytes, *n* = 3; **F**, **G** Immunofluorescent staining and quantification of P16 in knee cartilage of controls and DMM mice treated with vehicle, rmMFG-E8 or MFG-E8-NAb for 4 weeks or 8 weeks; *n* = 7 per group; Scale bar: 25 µm. ^*^P < 0.05, ^**^P < 0.01, ^***^P < 0.001, ^****^P < 0.0001, ns not significant.

Histomorphometric analysis revealed a progressive loss of articular cartilage in mice in a time-dependent manner after inhibition of MFG-E8. We then examined the effects of MFG-E8 in chondrocyte homeostasis. Significantly increased MMP13 and decreased Aggrecan (ACAN) and type 2 collagen (COL2) were observed in articular cartilage of mice treated with intraarticular injection of MFG-E8 neutralizing antibody (MFG-E8-NAb). However, recombinant mouse MFG-E8 (rmMFG-E8) supplementation reversed the increase in MMP13 expression and restored chondrocyte homeostasis by enhancing the release of proteoglycans (Fig. 3A–C and Fig. S3A, B). In vitro study in mouse primary chondrocytes showed that rmMFG-E8 enhanced the expression of anabolic markers and decreased catabolic factors, whereas MFG-E8 neutralizing antibody caused the opposite effects (Fig. S3C, D). Taken together, these data suggested that MFG-E8 regulated cartilage homeostasis by enhancing anabolism and inhibiting catabolism in chondrocytes.

### MFG-E8 defers chondrocyte senescence in OA

It is well-established that OA is an age-related disease and that cellular senescence is an important driving factor in OA pathogenesis. Hence, we explored the role of MFG-E8 in chondrocyte senescence during OA. β-galactosidase staining revealed that rmMFG-E8 markedly rescued the senescence phenotype induced by IL-1β in primary murine chondrocytes (Fig. 3D, E). Senescence-related hallmarks P16, P21 and P53 were significantly downregulated after ectopic application of MFG-E8 in IL-1β-pretreated primary chondrocytes, while MFG-E8 loss strongly promoted the expression of these senescence factors (Fig. S4A, B). Intraarticular injection of MFG-E8 neutralizing antibody obviously upregulated senescence markers, which were suppressed by rmMFG-E8, indicating the protective effect of MFG-E8 against chondrocyte senescence (Fig. 3F, G and Fig. S4C-D). Therefore, MFG-E8 may serve as a vital regulatory factor in chondrocyte senescence during OA development.

### MFG-E8 enhances M2 macrophage polarization and suppresses production of inflammatory cytokines in OA synovium

**Fig. 4:**
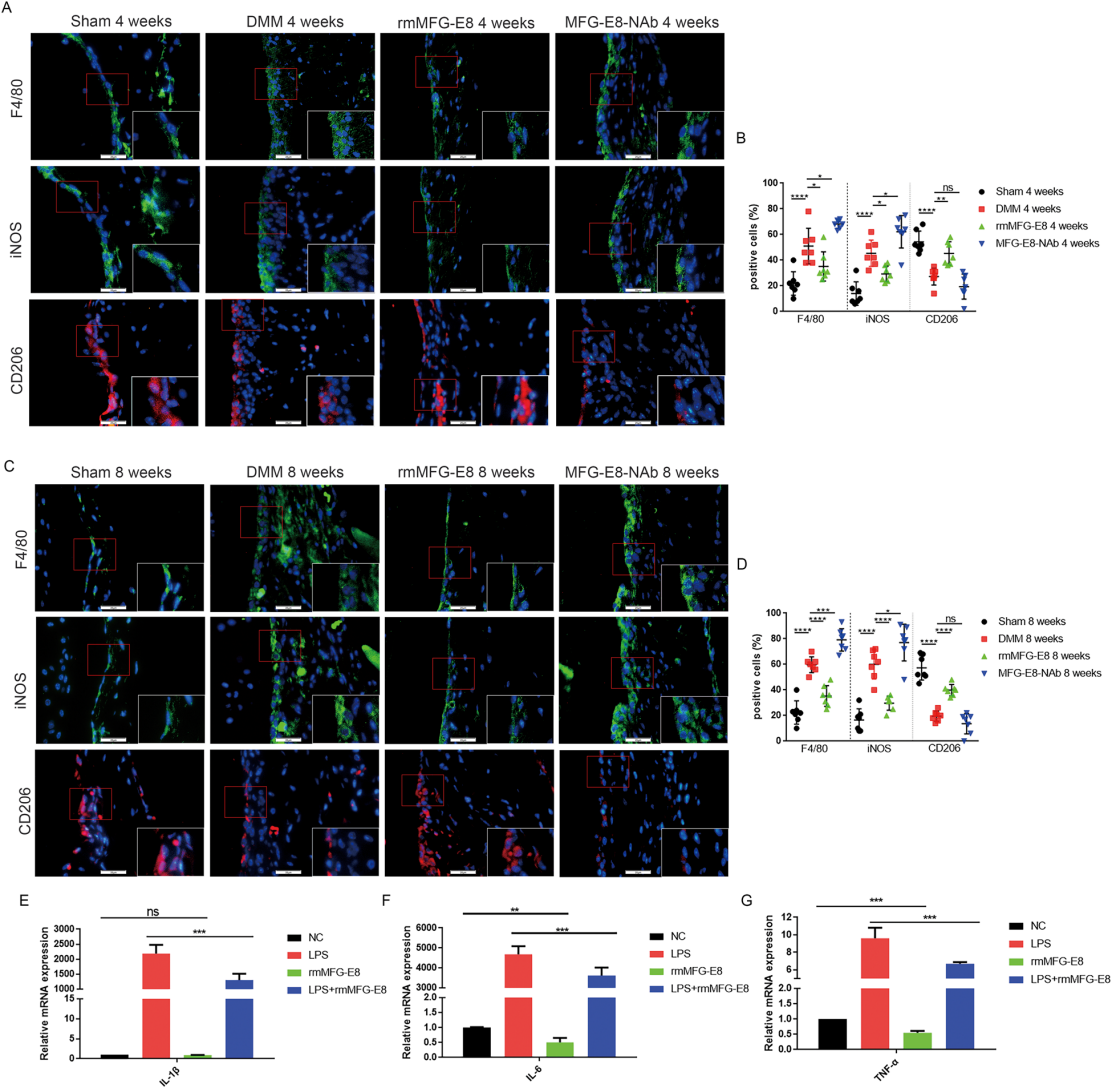
MFG-E8 enhances M2 macrophage polarization and suppresses expression of inflammatory cytokines in OA synovium. Immunofluorescent staining and quantification of F4/80, iNOS and CD206 in synovial tissue of sham-operated mice and mice treated with vehicle, rmMFG-E8 and MFG-E8-NAb for 4 weeks (**A**, **B**) or 8 weeks (**C**, **D**). *n* = 7 per group; Scale bar: 25 µm. Relative mRNA expression level of IL-1β (**E**), IL-6 (**F**) and TNF-α (**G**) in LPS- and rmMFG-E8-treated RAW264.7 cells, n = 3. ^*^P < 0.05, ^**^P < 0.01,^***^P < 0.001, ^****^P < 0.0001, ns not significant.

Recent research has underlined the role of synovial inflammation and synovial macrophage polarization in OA development. Considering that synovium thickness decreased after MFG-E8 administration, we hypothesized that MFG-E8 may influence synovitis progression via modulation of the macrophage subtype. Compared with controls, marked reductions in the number of F4/80 (macrophage marker)-positive cells were detected in rmMFG-E8-treated OA mice, together with a significant decrease of iNOS (M1-like macrophage marker)-positive cells. In contrast, the proportion of cells positive for the M2-like macrophage marker CD206 in rmMFG-E8-treated OA synovium showed a significant increase predominantly in the intimal lining layer both at 4 weeks and at 8 weeks post OA surgery, as well as at 14 days post CIOA treatment. Interestingly, upregulated F4/80 and iNOS were observed in MFG-E8 neutralizing antibody treated mouse synovium, while CD206-positive cells only showed a slight decrease (Fig. 4A–D and S5A, B). This finding was further confirmed in LPS- or IL-4 stimulated RAW264.7 cells (Fig. S5C, D). Moreover, MFG-E8 significantly restored the transcriptional upregulation of the proinflammatory factors IL-1β, IL-6 and TNF-α induced by LPS in RAW264.7 cells as indicated by quantitative reverse transcription PCR (qRT-PCR) (Fig. 4E–G). Moreover, ELISA validated the secretion of IL-1β was significantly inhibited by rmMFG-E8 in LPS treated bone-marrow derived macrophages (Fig. S5E). Together, these results suggested that MFG-E8 had the potential to reprogram macrophages and thus alleviate synovitis in OA.

### MFG-E8 suppresses OA progression through inhibition of the NF-κB pathway

**Fig. 5:**
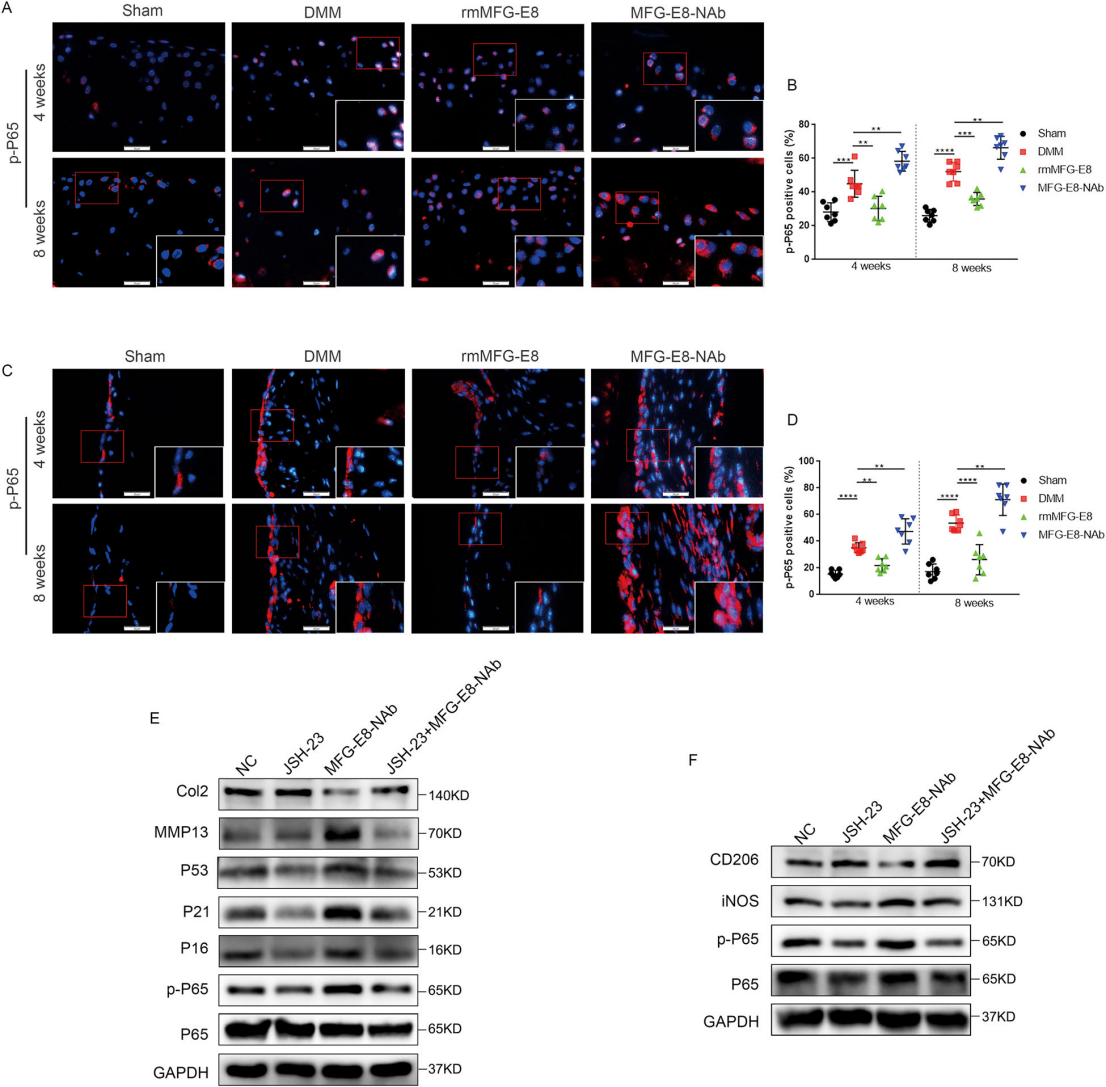
MFG-E8 suppresses OA progression through inhibition of the NF-κB pathway. Immunofluorescent staining and quantification of p-P65 in knee cartilage (**A**, **B**) and synovial tissue (**C**, **D**) of controls and mice administered vehicle, rmMFG-E8 or MFG-E8-NAb for 4 weeks or 8 weeks. *n* = 7 per group; Scale bar: 25 µm; **E** Immunoblotting of p-P65, P65, MMP13, Collagen II, and senescence markers (P16, P21, P53) of primary murine chondrocytes treated with JSH-23 or MFG-E8-NAb; **F** Immunoblotting of p-P65, P65, iNOS and CD206 of RAW264.7 cells treated with JSH-23 or MFG-E8-NAb. ^**^P < 0.01, ^***^P < 0.001^, ****^P < 0.0001.

We next sought to identify the MFG-E8-derived pathways responsible for chondrocyte senescence and macrophage reprogramming during OA. The NF-κB pathway is a well explored inflammatory pathway, which is over-activated in OA, exacerbating chondrocyte matrix degradation, synovial inflammation, and macrophage M1 polarization^21,22^. Excitingly, we found that MFG-E8 treatment rescued the phosphorylation of p65 NF-κB signaling, while neutralizing MFG-E8 significantly enhanced p65 phosphorylation both in cartilage and in synovium post OA surgery (Fig. 5A–D). In vitro studies in murine primary chondrocytes and RAW264.7 cells confirmed the suppressive effect of MFG-E8 in p65 NF-κB signaling (Fig. S6A–D). Furthermore, we determined whether NF-κB activation played a role in MFG-E8 deficiency-induced chondrocyte senescence and macrophage reprogramming. We found that the NF-κB pathway inhibitor JSH-23 partially reduced MFG-E8 loss-induced expression of P53, P21 and P16 in murine primary chondrocytes, and reduced the enhancing tendency of M1 macrophages caused by MFG-E8 deficiency (Fig. 5E, F). While NF-κB pathway activator phorbol 12-myristate 13-acetate (PMA) dismissed the inhibition of catabolism and senescence in chondrocytes, and decreased the M2 macrophage polarization induced by rmMFG-E8 (Fig. S6E, F). To sum up, the above results suggest that MFG-E8 loss causes chondrocyte senescence and inflammation, which may lead to articular cartilage degeneration and synovial hyperplasia, at least partly through p65 NF-kB signaling.

### MiR-99b-5p regulates MFG-E8 expression in OA

**Fig. 6:**
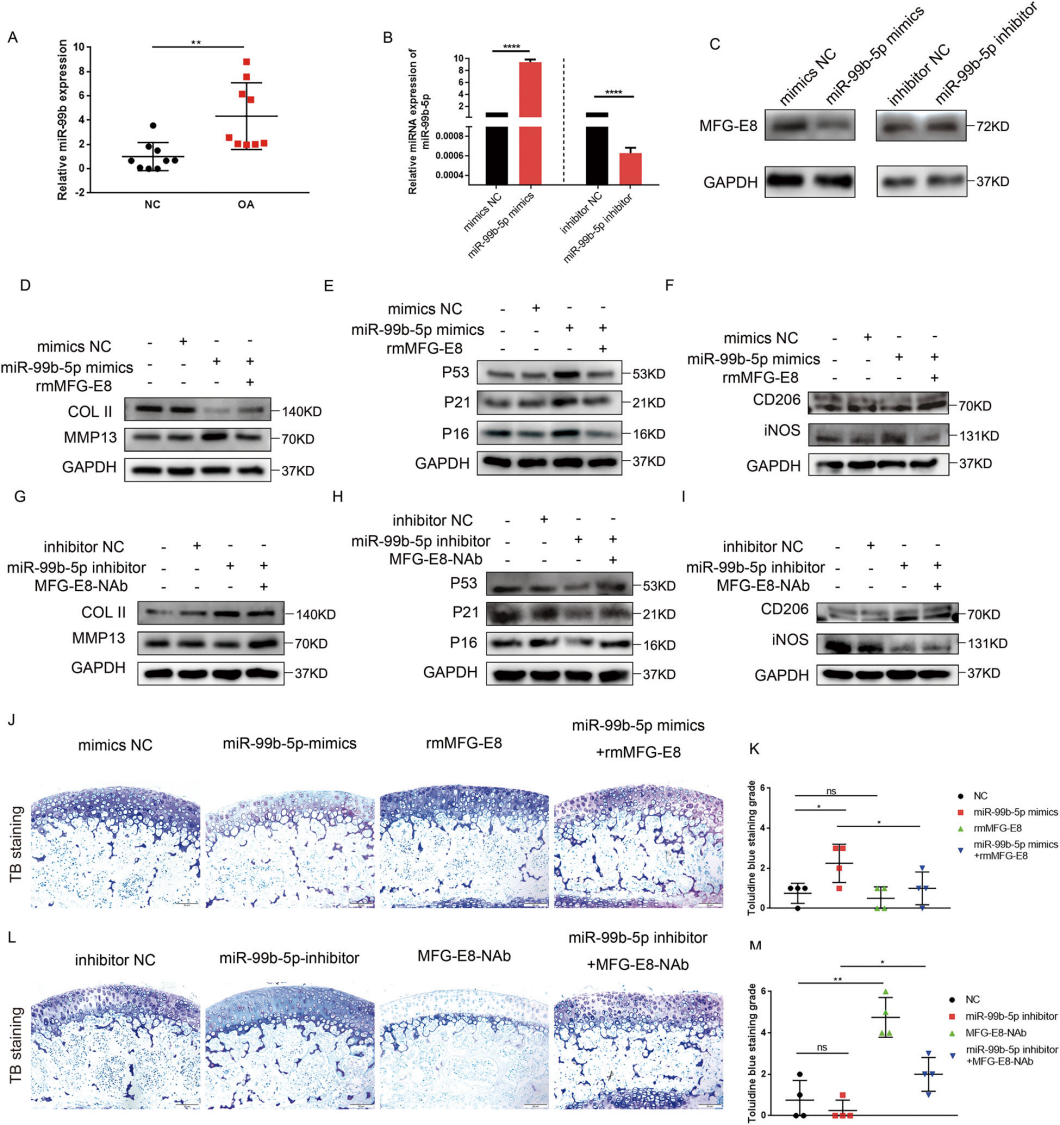
MiR-99b-5p regulates MFG-E8 expression in OA. **A** Relative expression of miR-99b-5p in articular cartilage of human knees from the lateral (NC) or medial (OA) tibial plateaus, *n* = 9 per group; **B** Relative expression of miR-99b-5p in primary murine chondrocytes transfected with miR-99b-5p mimics and inhibitor, *n* = 3; **C** Immunoblotting of MFG-E8 in primary murine chondrocytes treated with miR-99b-5p mimics and inhibitor; **D** Immunoblotting of Collagen II and MMP13 in miR-99b-5p mimics- or rmMFG-E8- treated primary murine chondrocytes; **E** Immunoblotting of senescence markers (P16, P21, P53) in miR-99b-5p mimics- or rmMFG-E8- treated primary murine chondrocytes; **F** Immunoblotting of iNOS and CD206 of RAW264.7 cells with miR-99b-5p mimics transfection or rmMFG-E8 administration; **G** Immunoblotting of Collagen II and MMP13 in miR-99b-5p inhibitor- or MFG-E8-NAb- treated primary murine chondrocytes; **H** Immunoblotting of senescence markers (P16, P21, P53) in miR-99b-5p inhibitor- or MFG-E8-NAb- treated primary murine chondrocytes; **I** Immunoblotting of iNOS and CD206 of RAW264.7 cells with miR-99b-5p inhibitor transfection or MFG-E8-NAb treatment; **J**, **K** Toluidine blue (TB) staining and grade of mouse tibial plateaus cartilage explants treated with miR-99b-5p mimics and/or rmMFG-E8; *n* = 4 per group; Scale bar: 50 µm; **L**, **M** TB staining and grade of mouse tibial plateaus cartilage explants treated with miR-99b-5p inhibitor and/or MFG-E8-NAb; *n* = 4 per group; Scale bar: 50 µm; ^*^P < 0.05,^**^P < 0.01, ^****^P < 0.0001, ns not significant.

The important role of microRNA in OA onset and progression has been widely studied. Hence, we aimed to investigate whether microRNA regulates the OA process through targeting MFG-E8. GSE126677, a dataset of miRNA sequences of synovial fluid-derived extracellular vesicles from three normal controls and two OA patients, was downloaded and analyzed to screen differentially-expressed microRNAs in OA. We identified 145 upregulated and 12 downregulated microRNAs (Fig. S7A), of which the top 20 upregulated and top 10 downregulated microRNAs are displayed in a heatmap (Fig. S7B). Among these miRNAs, miR-99b-5p was reported to be a direct binding factor of MFG-E8 mRNA as determined by luciferase reporter assay^23^. Hence, miR-99b-5p was selected for further investigation. In human OA knee cartilage, miR-99b-5p expression was significantly upregulated compared with the control samples (Fig. 6A). Next, successful overexpression or inhibition, respectively, of miR-99b-5p in mimic- or inhibitor-transfected primary murine chondrocytes was confirmed (Fig. 6B). We found inhibited expression of MFG-E8 in cells treated with miR-99b-5p mimics and upregulated expression in cells treated with miR-99b-5p inhibitor (Fig. 6C). We then explored the role of miR-99b-5p in OA development. Increased MMP13 and decreased COL2 were detected in mimics-treated chondrocytes, accompanied by enhanced senescence-related hallmarks P16, P21, and P53 (Fig. 6D, E). Meanwhile, elevated iNOS and suppressed CD206 expression were verified in mimics-transfected RAW264.7 cells (Fig. 6F). These effects of miR-99b-5p could be markedly diminished by exogenous use of MFG-E8 (Fig. 6D–F). Accordingly, miR-99b-5p inhibitor rescued the enhanced MMP13, decreased type 2 collagen, increased senescence-related factors induced by IL-1β in primary chondrocytes, as well as reversing upregulated iNOS and suppressing CD206 in LPS-stimulated RAW264.7 cells (Fig. 6G–I). These phenotypes were likely counteracted by MFG-E8 neutralization (Fig. 6G–I). To verify the effects of miR-99b-5p in cartilage tissue, cartilage explants were obtained from 3-week-old male C57 mice. Loss of toluidine blue staining was observed in cartilage explant administered with miR-99b-5p mimics, while this damage was reversed with rmMFG-E8 treatment in part (Fig. 6J, K). Consistently, no obvious proteoglycan loss was found in miR-99b-5p inhibitor treated cartilage explant, whereas MFG-E8 neutralization diminished this protective effect (Fig. 6L, M). Altogether, these data demonstrated that miR-99b-5p accelerated OA progression by directly binding and degrading MFG-E8 mRNA.

## Discussion

**Fig. 7:**
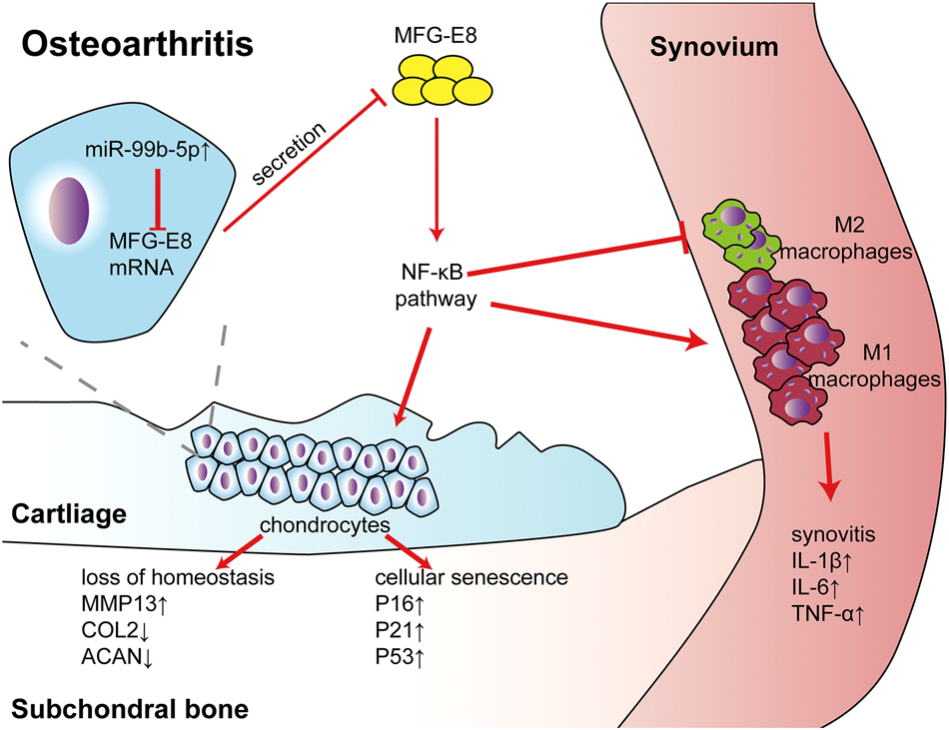
Model of MFG-E8 secreted by chondrocytes in modulating chondrocyte homeostasis, senescence, and synovial macrophage polarization during OA. Increased miR-99b-5p inhibited MFG-E8 expression and secretion in OA, while decreased MFG-E8 disrupted chondrocyte homeostasis, promoted chondrocyte senescence, and unbalanced synovial macrophage polarization through NF-κB signaling, thus accelerated OA progression.

In this study we demonstrated for the first time that MFG-E8 is a key factor mediating chondrocyte senescence and macrophage polarization during the pathogenesis and progression of OA. We showed that MFG-E8 expression was markedly downregulated by miR-99b-5p, which in turn exacerbated cartilage degeneration, synovial inflammation, and osteophyte formation during OA partly through NF-κB signaling (Fig. 7). Our findings demonstrated a functional pathway important for OA development and identified intra-articular injection of MFG-E8 as a potential therapy for OA prevention and treatment.

Aging has long been recognized as a major risk factor for OA. Evidence showed that senescent chondrocytes (SnCs) aggregate with age and are markedly upregulated in human OA cartilage compared with healthy controls^24^. Moreover, excessive SnCs lead to severer articular cartilage destruction, while SnC clearance alleviates subsequent senescence and tissue damage of surrounding cells, thereby delaying OA progression^24,25^. Besides, senescent chondrocytes release massive SASP molecules capable of digesting ECM and triggering further inflammation, which is deemed as the main driving factor of senescence induced OA progression rather than restrained chondrocyte replication^26^. These findings suggest a strong correlation between senescence and severity of OA, but the modulating mechanism of SnCs in OA remains obscure. In our study, we found MFG-E8 significantly downregulated SA-β-gal staining-positive cells. Similarly, enhanced SnC markers, including p16, p53 and p21 in articular cartilage of DMM mice were effectively reversed with rmMFG-E8 supplement. These results suggest MFG-E8 regulates subculture-induced chondrocyte senescence, which might be associated with OA pathogenesis.

Accumulating evidence demonstrates that an imbalance of M1/M2 macrophage polarization plays an essential role in OA inflammation, which has been in the spotlight recently^11^. Besides assisting the clearance of damaged cells, MFG-E8 also plays an important role in inflammation. Hansen et al. found that in neonatal sepsis lung injury, the levels of inflammatory factors, including IL-6 and MIP-2, as well as neutrophil infiltration into the lung, were significantly reduced by recombinant MFG-E8 administration^16^. Another essential finding regarding MFG-E8 was that it could reprogram macrophages to an anti-inflammatory and pro-repair M2 subtype^27^. One study showed that the number of M2 macrophages and the M2 macrophage/total macrophage ratio in wounds significantly increased after injection of MFG-E8 WT MSCs compared to MFG-E8 KO MSCs^15^. However, whether MFG-E8 regulated these phenotypes in OA was still unclear. For the first time, we confirmed that MFG-E8 deficiency in OA led to an imbalance between anabolism and catabolism in articular cartilage, as well as skewing synovial macrophage polarization towards the proinflammatory M1 subtype. Meanwhile exogenous administration of MFG-E8 had the ability to convert M1 polarized macrophages into M2 macrophages and to decrease inflammatory cytokine release during OA development. MFG-E8 may attenuate OA progression partially by prevention of pathological M1 macrophage polarization and accumulation of compensatory M2 macrophages, indicating that MFG-E8 mediates macrophage reprogramming in OA.

Emerging findings are elucidating the essential role of NF-κB signaling in OA progression. Aberrant activation of the NF-κB pathway not only stimulates the transcription of catabolic genes such as MMP1, MMP9, and ADAMTS5, but also induces the release of inflammatory mediators such as IL-1β, IL-6, and TNF-α through a positive feedback loop, which further exacerbates cartilage destruction^28^. In synovial tissues, NF-κB increased 10-fold in early OA, which leads to excessive production of inflammatory cytokines, catabolic modulators, and angiogenic factors^29^. Moreover, NF-κB signaling induces premature cellular senescence, whereas NF-κB inhibitors and NF-κB p65 siRNA remarkably suppress the expression of P21 and P53^30^. Recent studies also highlighted a strong link between the NF-κB pathway and enhanced M1 macrophage polarization^31,32^. In this study, we further confirmed that NF-κB signaling was involved in OA-associated phenotype. More importantly, NF-κB pathway inhibitor remarkably restored chondrocyte catabolism, senescence, and aberrant synovial macrophage polarization caused by MFG-E8 neutralization, while NF-κB pathway activator counteracted the protective effects of MFG-E8 supplementation, indicating that MFG-E8 protected against OA partially through the NF-κB pathway.

It is well accepted that microRNA may play a pivotal role in regulating OA onset and development. The mechanisms of several microRNAs modulating OA pathology are well studied, such as miR-27b, miR-181b, miR-21, and miR-142–3p^33^. We speculated that the decrease of MFG-E8 in OA may be caused by aberrant expression of certain microRNAs. Among the differentially-expressed microRNAs screened out in OA synovial fluid, miR-99b-5p was found to be upregulated in OA and has been confirmed to directly bind MFG-E8 in another study^23^. The roles and mechanisms of miR-99b-5p in other diseases have been widely reported. In acute lung injury, NF-κB (p65) promotion of miR-99b-5p accelerates the disease progression^34^. In tumor-associated phenotypes, miR-99b-5p promotes M1 while suppressing M2 subtype polarization^35^. Moreover, overexpression of miR-99b-5p downregulates protein synthesis in muscle^36^. In this study, we validated the role of miR-99b-5p in OA pathogenesis. Higher levels of miR-99b-5p were detected in OA cartilage, and overexpression of miR-99b-5p led to suppressed cartilage anabolism, accelerated chondrocyte senescence, and aberrant synovial macrophage polarization, which indicated its role in OA exacerbation. Interestingly, exogenous use of MFG-E8 effectively rescued these deleterious effects, suggesting that miR-99b-5p may take effect in OA by binding and inhibiting MFG-E8.

Potential limitations of our research include the use of intra-articular recombinant murine MFG-E8 protein and MFG-E8 neutralizing antibody injection rather than the use of transgenic mice, and the role of MFG-E8 in relieving pain and regaining joint function in OA is worth exploring in future study. Moreover, we noticed an interesting fact that MFG-E8 was significantly downregulated in OA articular chondrocytes, while upregulated in the hypertrophic and ossified chondrocytes of meniscus and osteophytes. We speculate it is because permanent phenotypic chondrocytes (articular chondrocytes) and transient phenotypic chondrocytes (meniscal and osteophytic chondrocytes) have different gene expression patterns^37,38^. Since MFG-E8 is an important osteogenesis regulator and lack of MFG-E8 leads to impaired bone formation and increased bone loss as previously reported, chondrocytes of meniscus and osteophyte may overexpress MFG-E8 to facilitate their ossification, namely to display the transient chondrocyte phenotype^39,40^. Hence MFG-E8 might be a potential biomarker to distinguish chondrocytes with transient or permanent phenotypes. Besides, in other diseases, MFG-E8 alleviates apoptosis and inflammation, while enhances M2 polarization by binding to its receptor integrin αVβ3^41,42^. Interaction between MFG-E8 and integrin αVβ3 might be a promising therapeutic target in OA, which needs to be further studied.

In summary, our findings broadened the potential clinical application of MFG-E8. MFG-E8 loss due to miR-99b-5p in chondrocytes initiated and promoted OA development by targeting chondrocyte senescence and macrophage reprogramming to stimulate cartilage degeneration, synovial inflammation and osteophyte formation. Targeting MFG-E8 by intraarticular supplementation represents an approach to delay OA development.

## Materials and methods

### Human samples

After the study was approved by the Ethics Committee of the Third Affiliated Hospital of Southern Medical University, ten tibial plateaus were obtained from OA patients who were undergoing total knee replacement surgery after obtaining informed consent. The medial part of each tibial plateau was used as the OA group, while the lateral tibial plateau with less cartilage destruction was implemented as the control group.

### Mice

We purchased 12-week-old male C57BL/6J (wild-type) mice from the Experimental Animal Centre of Southern Medical University (Guangzhou, China) and mice were raised to 14-week-old before surgery. Importation, transportation and housing of the mice were all conducted according to the recommendations of “The use of non-human primates in research.” All mice were maintained in accordance with institutional animal care and use guidelines. Surgery to destabilize the medial meniscus (DMM) was performed on the right knee of 14-week-old mice to surgically create a mouse model of induced OA. Sham surgery was performed by opening and exposing the tissues of the right knee and then suturing the incision without intervention of the meniscus in age-matched mice. Before surgery, mice were anesthetized with pentobarbital through intraperitoneal injection. After the surgery, 3 µL PBS (vehicle)/mouse, 50 ng/g recombinant mouse MFG-E8 (rmMFG-E8, R&D systems, Minneapolis, MN, USA, #2805-MF) or 150 ng/g MFG-E8 neutralizing antibody (MFG-E8-NAb, MBL, Nagoya, Japan, #D161–3) was administered by intra-articular injection once per week. The right legs were harvested 4 or 8 weeks post-surgery (*n* = 7 in each group). Experimental collagenase-induced OA (CIOA) was induced in 12-week-old male C57 mice as previously described^43^. Briefly, the right knee joint of each mouse was intraarticularly injected with 1 unit of type VII collagenase (Sigma-Aldrich, St. Louis, MO, USA) dissolved in 5 µL physiologic saline on day 0 and 2, with 5 µL saline injection mice as the control group. 3 µL PBS, 50 ng/g rmMFG-E8, or 150 ng/g MFG-E8-NAb were delivered by intra-articular injection 7 days after the first dose of collagenase. The right legs were obtained on days 14 after collagenase treatment (*n* = 6 in each group). Then samples were fixed, decalcified, dehydrated and sectioned. The Southern Medical University Animal Care and Use Committee approved all procedures involving mice.

### Cartilage explants

Three-week-old male C57 mice were euthanized to isolate tibial plateaus cartilage explants. Microforceps were used to blunt dissect cartilage from underlying bone. Explants were cultured for 3 days in DMEM/F12 containing 10% fetal bovine serum in 96-well plates before further processing.

### Cells

Primary murine chondrocytes were derived from tibial plateaus of 6-day-old C57 mice according to the protocol described previously^44^, and were cultured in maintenance medium consisting of Dulbecco’s modified Eagle’s medium (DMEM) nutrient mixture F12 (DMEM:F12) (Gibco, Carlsbad, CA, USA) supplemented with 20% fetal bovine serum (FBS) (Gibco) and 1% Penicillin-Streptomycin. Mouse macrophage-like RAW264.7 cells (ATCC, Manassas, VA, USA) were grown and maintained in DMEM with 10% FBS. Bone marrow derived macrophages (BMDM) were obtained from bone marrow of 6-week-old female C57 mice. The femurs and tibias were collected after the mice were sacrificed. The bone marrow cavities were flushed with complete DMEM containing 10% fetal bovine serum. After red cells were depleted, the remained cells were cultured for 16 h. Non-adherent cells were collected and planted in the complete DMEM containing 30 ng/mL M-CSF (R&D) for 72 h to induce BMDMs. Primary murine chondrocytes were treated with 30 ng/mL interleukin-1β (IL-1β) (R&D systems) for 24 h to create an in vitro OA chondrocyte model. RAW 264.7 cells and BMDMs were treated with 500 ng/mL lipopolysaccharide (LPS) (Invitrogen, San Diego, CA, USA) for 24 h to induce M1 polarization. RAW 264.7 cells were treated with 20 ng/mL IL-4 (R&D systems) for 24 h to induce M2 polarization. Primary chondrocytes, RAW264.7 cells, or BMDMs were treated with 500 ng/mL rmMFG-E8, 1500 ng/mL MFG-E8-NAb, 30 μM NF-κB inhibitor JSH-23 (MCE, Monmouth Junction, NJ, USA), or 10 ng/mL NF-κB activator PMA (MCE).

### The qRT-PCR

Total RNA was isolated from primary murine chondrocytes, RAW264.7 cells, and ground cartilage from human tibial plateaus using TRIzol reagent (Takara Bio Inc., Shiga, Japan). For mRNA quantification, 1 mg of total RNA was purified with genomic DNA (gDNA) remover and reverse transcribed using 5× HiScript II qRT SuperMix II (Vazyme Biotech, Nanjing, China). Each PCR reaction consisted of 10 µL 2× ChamQ SYBR qPCR Master Mix (Vazyme), 10 µM forward and reverse primers, and 500 ng of cDNA. For miRNA quantification, 1 mg total RNA was purified with gDNA wiper mix and then reverse transcribed using Hiscript II Enzyme Mix, 10× RT Mix, and specific stem-loop primers. Template DNA was mixed with 2× miRNA Universal SYBR qPCR Master Mix, specific primers and mQ primer R (Vazyme). All reactions were run in triplicate. Primer sequences were of mice and were listed below: MFG-E8 Forward 5’-CCG CGT CTG GTG ACT TCT G-3’, Reverse 5′-TCC TCT CTC AGT CTC ATT GCAC-3’, iNOS Forward 5′-GTT CTC AGC CCA ACA ATA CAA GA-3’, Reverse 5’-GTG GAC GGG TCG ATG TCA C-3’, GAPDH forward 5′-AAA TGG TGA AGG TCG GTG TGA AC-3’, reverse 5′-CAA CAA TCT CCA CTT TGC CAC TG-3’, CD206 forward 5′-CTC TGT TCA GCT ATT GGA CGC-3’, reverse 5′-TGG CAC TCC CAA ACA TAA TTT GA-3’, IL-1β forward 5′-GCA ACT GTT CCT GAA CTC AAC T-3’, reverse 5′-ATC TTT TGG GGT CCG TCA ACT-3’, IL-6 forward 5′-ACA ACC ACG GCC TTC CCT ACT T-3’, reverse 5′-CAG GAT TTC CCA GCG AAC ATG TG-3’,TNF-α forward 5′-CCT CCC TCT CAT CAG TTC TA-3’, reverse 5′-ACT TGG TTT GCT ACG AC-3’, (hsa-/mmu-) miR-99b-5p stem-loop primer 5′-GTC GTA TCC AGT GCA GGG TCC GAG GTA TTC GCA CTG GAT ACG ACC GCA AG-3’, forward primer 5′-CGC ACC CGT AGA ACC GAC-3’.

### Western blot analysis

Cells cultured in 12-well dishes were lysed with 100 μL of radioimmunoprecipitation assay (RIPA) buffer (Beyotime Institute of Biotechnology, Jiangsu, China) containing protease inhibitor and phosphatase inhibitor. Proteins were subjected to sodium dodecyl sulfate-polyacrylamide gel electrophoresis (SDS-PAGE), and then were transferred to polyvinylidene difluoride (PVDF) membranes (Beyotime). After incubation with 5% skim milk in 50 mM Tris-buffered saline (TBS) (pH 7.4) containing 0.1% Tween-20 (TBST) for 1 h at room temperature, the membranes were further incubated overnight at 4 °C with primary antibodies diluted with 5% BSA TBST. Following three washes with TBST (5 min each), the membranes were incubated with secondary antibodies (diluted at 1:3000 in TBST) for 1 h at room temperature. Target protein bands were visualized by FDbio-Dura ECL (FDbio science, Hangzhou, China). Antibodies used for western blotting were: goat anti-MFG-E8 (R&D Systems, 1:1,000, AF2805), rabbit anti-MMP13 (Proteintech, Rosemont, IL, USA, 1:1,000, 18165-1-AP), rabbit anti-Collagen II (COL2) (Abcam, Cambridge, UK, 1:1,000, ab34712), rabbit anti-P16 (Abcam, 1:1,000, ab51243), rabbit anti-P21 (Abcam, 1:1,000, ab188224), rabbit anti-P53 (Proteintech, 1:1,000, 10442-1-AP), rabbit anti-phosphorylated P65 (p-P65) (CST, Danvers, MA, USA, 1:1,000, 3033), rabbit anti-P65 (CST, 1:1,000, 8242), mouse anti-iNOS (Immunoway, Plano, TX, USA, 1:1,000, YT3169), mouse anti-CD206 (Abcam, 1:1,000, ab64693), species-matched horseradish peroxidase-conjugated secondary antibodies (Jackson ImmunoResearch Laboratories, West Grove, PA, USA).

### Enzyme-linked immunosorbent assay (ELISA)

Mouse blood was collected 4 weeks and 8 weeks post sham or DMM surgery via intracardiac puncture and placed in non-EDTA-containing microcentrifuge tubes. All blood samples were sedimented for at least 1 h at room temperature, then spun down at 4500 × *g* for 10 min, and serum was collected into new microcentrifuge tubes. The culture supernatant from primary murine chondrocytes treated with or without IL-1β was collected after 24 h. Samples were stored at −80 °C until use. A mouse MFG-E8 Quantikine Kit (R&D Systems) was used to measure the concentration of MFG-E8 in mouse serum and chondrocyte culture supernatant. The culture supernatant from BMDMs administered with or without 500 ng/mL LPS or 500 ng/mL rmMFG-E8 was harvested 24 h after treatment. Samples were stored at −80°C. A mouse IL-1β Quantikine Kit (Meimian, China) was applied to test the IL-1β level in BMDM culture supernatant.

### Histology and immunohistochemical (IHC)/immunofluorescence (IF) staining

Knee joint tissues were fixed in 4% paraformaldehyde for 48 h, decalcified for 21 days, then dehydrated and embedded in paraffin. Serial mid-sagittal sections (4 μm thick) were cut and stained with Safranin O-fast green/hematoxylin and eosin (H&E)/toluidine blue for morphological analysis. IHC and IF staining were performed on the 4-μm thick tissue sections. Slides were deparaffinized, rehydrated, and washed three times in PBS for 5 min each. Antigen retrieval was performed by soaking slides in citric acid in a 60 °C water bath overnight. After washing three times in PBS, slides were then quenched in 3% hydrogen peroxide for 10 min at room temperature and washed with PBS another three times. Then slides were blocked with 10% normal bovine serum (Solarbio, Beijing, China) for 1 h at room temperature. Slides were then incubated with primary antibodies at 4 °C overnight. The secondary antibody for IHC or fluorescent secondary antibody for IF were applied for 1 h at room temperature, then IHC slides were stained with DAB and hematoxylin, dehydrated and mounted. IF slides were processed with 4, 6-diamidino-2-phenylindole (DAPI, Thermo Fisher Scientific, Waltham, MA, USA) staining solution and mounted. Antibodies used for IHC/IF staining were: rabbit anti-MFG-E8 (Abclonal, Woburn, MA, USA, 1:100, A12322), rabbit anti-MMP13 (Proteintech, 1:400, 18165-1-AP), rabbit anti-Aggrecan (Proteintech, 1:400, 13880-1-AP), rabbit anti-COL2 (Abcam, 1:100, ab34712), mouse anti-F4/80 (Santa Cruz Biotechnology, 1:100, sc-377009), mouse anti-iNOS (Santa Cruz Biotechnology, 1:100, sc-7271), mouse anti-CD206 (Proteintech, 1:100, 18704-1-AP), mouse anti-P16 (Abcam, 1:400, ab51243), mouse anti-P21 (Abclonal, 1:400, A1483), mouse anti-phosphorylated P65 (p-P65) (CST, 1:100, 3033), species-matched horseradish peroxidase-conjugated secondary antibodies (Jackson ImmunoResearch Laboratories), species-matched Alexa-488 or -594-labeled secondary antibody (Life Technologies, Carlsbad, CA, USA).

### Grading of cartilage and synovium structure

Histology sections of the knee joints were graded by two blinded observers based on the Osteoarthritis Research Society International (OARSI) scoring system developed by Glasson et al^45^. Generally, sections were assigned a grade of 0–6: 0, normal cartilage; 0.5, slight loss of Safranin O staining without structural changes; 1, small fibrillations without loss of cartilage; 2, vertical clefts down to the layer below the superficial layer; 3–6, vertical clefts or erosion to the calcified cartilage affecting <25% (grade 3), 25–50% (grade 4), 50–75% (grade 5) and >75% (grade 6) of the articular surface. Toluidine blue staining of cartilage explants were graded by two blinded observers based on the area of staining loss by using a 6-point scale^46^. Synovitis severity was estimated by two blinded observers based on the enlargement of the synovial lining cell layer, density of the resident cells, and inflammatory infiltration. A 9-point scale was used with low scores indicating moderate synovitis while high scores represented severe synovitis^47^.

### Micro-CT analysis

Micro-computed tomography (micro-CT) of fixed knee joint specimens was performed using a microtome imaging system (ZKKS-MCT-Sharp-III scanner, Caskaishen, China). A small field was selected for scanning and corrected for the CT value, with a 70 kV scanning voltage, 30 W power, 429 μA current and 5 μm scan thickness. The software 3D-MED 3.0 was used for three-dimensional knee reconstruction and image capture. We defined the region of interest to cover all the osteophytes, and total bone volume (BV) of osteophytes was then analyzed.

### Senescence-associated β-galactosidase staining

Senescence-associated β-galactosidase (SA-βGal) activity was measured using a staining kit (Beyotime). Chondrocytes were plated into wells of a 12-well plate and fixed for 10 min at room temperature, washed, and incubated with the staining solution overnight at 37 °C.

### miR-99b-5p mimics and inhibitor transfection

Thirty nanomolar miR-99b-5p mimics or mimics control (GenePharma, Suzhou, China) were transfected into primary murine chondrocytes and RAW264.7 cells using lipofectamine 3000 (2.5 μL/mL) (Thermo Fisher Scientific) for 48 h following the manufacturer’s protocols. Fifty nanomolar miR-99b-5p inhibitor or inhibitor control (GenePharma, Suzhou, China) were transfected with Lipofectamine 3000 into primary murine chondrocytes treated with 30 ng/mL IL-1β and RAW264.7 cells treated with 500 ng/mL LPS. Then the cells were processed with Trizol for RNA analysis or RIPA for western blot analysis as described above. Cartilage explants were transfected with 1 µM miR-99b-5p (or control) mimics or inhibitor for 72 h following the guidance of previous researches^48^. Moreover, then samples were fixed, decalcified, dehydrated, and sectioned.

miR-99b-5p mimics F: 5ʹ-CACCCGUAGAACCGACCUUGCG-3ʹ, R: 5ʹ-CAAGGUCGGUUCUACGGGUGUU-3ʹ;miR-99b-5p inhibitor: 5′-CGCAAGGUCGGUUCUACGGGUG -3′;mimics NC: 5′-UUCUUCGAACGUGUCACGUTT-3′, R: 5′-ACGUGACACGUUCGGAGAATT-3′;inhibitor NC: 5′-CAGUACUUUUGUGUAGUACAA-3′.

### Bioinformatic analysis

GSE126677^49^, a dataset of miRNA sequences of synovial fluid-derived extracellular vesicles from three normal and two OA patients, was downloaded from the GEO platform. Data were normalized and transformed into regularized log (rlog) format. The Limma package was used to analyze the differentially-expressed microRNAs with the log fold change cutoff set as 1.5. The ggplot2 package and pheatmap package were used to draw a volcano plot and heatmap.

### Statistical analyses

Data are presented as the mean ± SD. For experiments comparing two groups of data, unpaired Student’s *t*-test was performed. For data involving multiple groups, one-way analysis of variance (ANOVA) was performed followed by Turkey’s post-hoc test. *P*-values < 0.05 were considered significant.

## Supplementary information

Supplementary Figure Legends

Figure S1

Figure S2

Figure S3

Figure S4

Figure S5

Figure S6

Figure S7

## Data Availability

All data generated or analyzed during this study are included in this submitted article and its additional files.
