# Supplementary material for: MFG-E8 regulated by miR-99b-5p protects against osteoarthritis by targeting chondrocyte senescence and macrophage reprogramming via the NF-κB pathway
Source: Cell Death Dis. 2021 May 25;12(6):533. doi: 10.1038/s41419-021-03800-x (PMC8144578; doi:10.1038/s41419-021-03800-x)
Supplement: Supplementary file 1 — Supplementary Figure Legends [file 41419_2021_3800_MOESM1_ESM.docx]

Figure S1. (A) Relative mRNA expression level of MFG-E8 in IL-1β-treated primary murine chondrocytes. n=3; (B) Immunoblotting of MFG-E8 in IL-1β-treated primary murine chondrocytes; (C) ELISA of MFG-E8 in culture supernatant of controls and IL-1β-treated primary murine chondrocytes. n = 8 per group. (D, E) Immunostaining and quantification of MFG-E8 of articular synoviocytes from controls and OA model mice at 4 weeks and 8 weeks post DMM operations; Scale bar: 100 µm; n=7 per group; (F) Immunoblotting of MFG-E8 and iNOS in bone marrow derived macrophages (BMDM) treated with or without LPS; ^**^*P* < 0.01, ^***^*P* < 0.001.

Figure S2. (A) Toluidine blue staining of articular cartilage from controls and mice treated with vehicle, rmMFG-E8 or MFG-E8-NAb for 4 weeks or 8 weeks. n=7 per group; Scale bar: 50 µm; (B) H&E staining of articular cartilage from controls and mice treated with vehicle, rmMFG-E8 or MFG-E8-NAb for 4 weeks or 8 weeks. Scale bar: 50 µm; (C) Hyaline cartilage/Calcified cartilage (HC/CC) ratio of the joints described in (B). n = 7 per group; (D, E) H&E staining of synovial tissues from controls and CIOA mice treated with vehicle, rmMFG-E8 and MFG-E8-NAb for 14 days. Scale bar: 100 µm; n=6 per group; ^*^*P* < 0.05, ^**^*P* < 0.01, ^****^*P* < 0.0001.

Figure S3. (A, B) Immunohistochemical staining and quantification of Aggrecan (ACAN) in knee cartilage from controls and DMM mice treated with vehicle, rmMFG-E8 and MFG-E8-NAb for 4 weeks or 8 weeks. n = 7 per group; Scale bar: 200 µm; (C) Immunoblotting of COL2 and MMP13 in IL-1β- and rmMFG-E8-treated primary murine chondrocytes; (D) Immunoblotting of COL2 and MMP13 in IL-1β- and MFG-E8-NAb-stimulated primary murine chondrocytes. ^*^*P* < 0.05, ^**^*P* < 0.01, ^****^*P* < 0.0001.

Fig S4. (A) Immunoblotting of P16, P21 and P53 in IL-1β- and rmMFG-E8-treated primary murine chondrocytes; (B) Immunoblotting of P16, P21 and P53 in IL-1β- and MFG-E8-NAb-stimulated primary murine chondrocytes; (C, D) Immunofluorescence staining and quantification of P21 in knee cartilage from controls and DMM mice treated with vehicle, rmMFG-E8 and MFG-E8-NAb for 4 weeks or 8 weeks. n = 7 per group; Scale bar: 25 µm; ^*^*P* < 0.05, ^**^*P* < 0.01, ^***^*P* < 0.001, ^****^*P* < 0.0001.

Figure S5. (A,B) Immunofluorescent staining and quantification of F4/80, iNOS and CD206 in synovial tissue of control mice and CIOA-mice treated with vehicle, rmMFG-E8 and MFG-E8-NAb for 14 days; Scale bar: 25µm; n=6 per group; (C) Relative mRNA expression of iNOS in LPS- and rmMFG-E8-treated RAW264.7 cells. n=3; (D) Relative mRNA expression of CD206 in IL-4- and rmMFG-E8-treated RAW264.7 cells; (E) ELISA of IL-1β in culture supernatant of controls, LPS-, rmMFG-E8-, and rmMFG-E8+LPS- treated BMDM; n=6 per group; ^*^*P* < 0.05, ^**^*P* < 0.01, ^***^*P* < 0.001, ^****^*P* < 0.0001，ns not significant.

Figure S6. (A) Immunoblotting of p-P65 and P65 of IL-1β- and rmMFG-E8-treated primary chondrocytes; (B) Immunoblotting of p-P65 and P65 of primary murine chondrocytes treated with IL-1β and MFG-E8-NAb; (C) Immunoblotting of p-P65 and P65 of LPS- and rmMFG-E8-treated RAW264.7 cells; (D) Immunoblotting of p-P65 and P65 of IL-1β- and MFG-E8-NAb-treated RAW264.7 cells; (E) Immunoblotting of p-P65, P65, MMP13, Collagen II, and senescence markers (P16, P21, P53) of primary murine chondrocytes treated with PMA or rmMFG-E8; (F) Immunoblotting of p-P65, P65, iNOS and CD206 of RAW264.7 cells treated with PMA or rmMFG-E8.

Figure S7. (A) Differentially-expressed microRNA in synovial fluid-derived extracellular vesicles from normal controls or OA patients based on GSE126677; (B) Twenty upregulated and ten downregulated microRNAs from synovial fluid-derived extracellular vesicles from controls and OA patients based on GSE126677.
