# Supplementary figures and images for: MFG-E8 regulated by miR-99b-5p protects against osteoarthritis by targeting chondrocyte senescence and macrophage reprogramming via the NF-κB pathway
Source: Cell Death Dis. 2021 May 25;12(6):533. doi: 10.1038/s41419-021-03800-x (PMC8144578; doi:10.1038/s41419-021-03800-x)

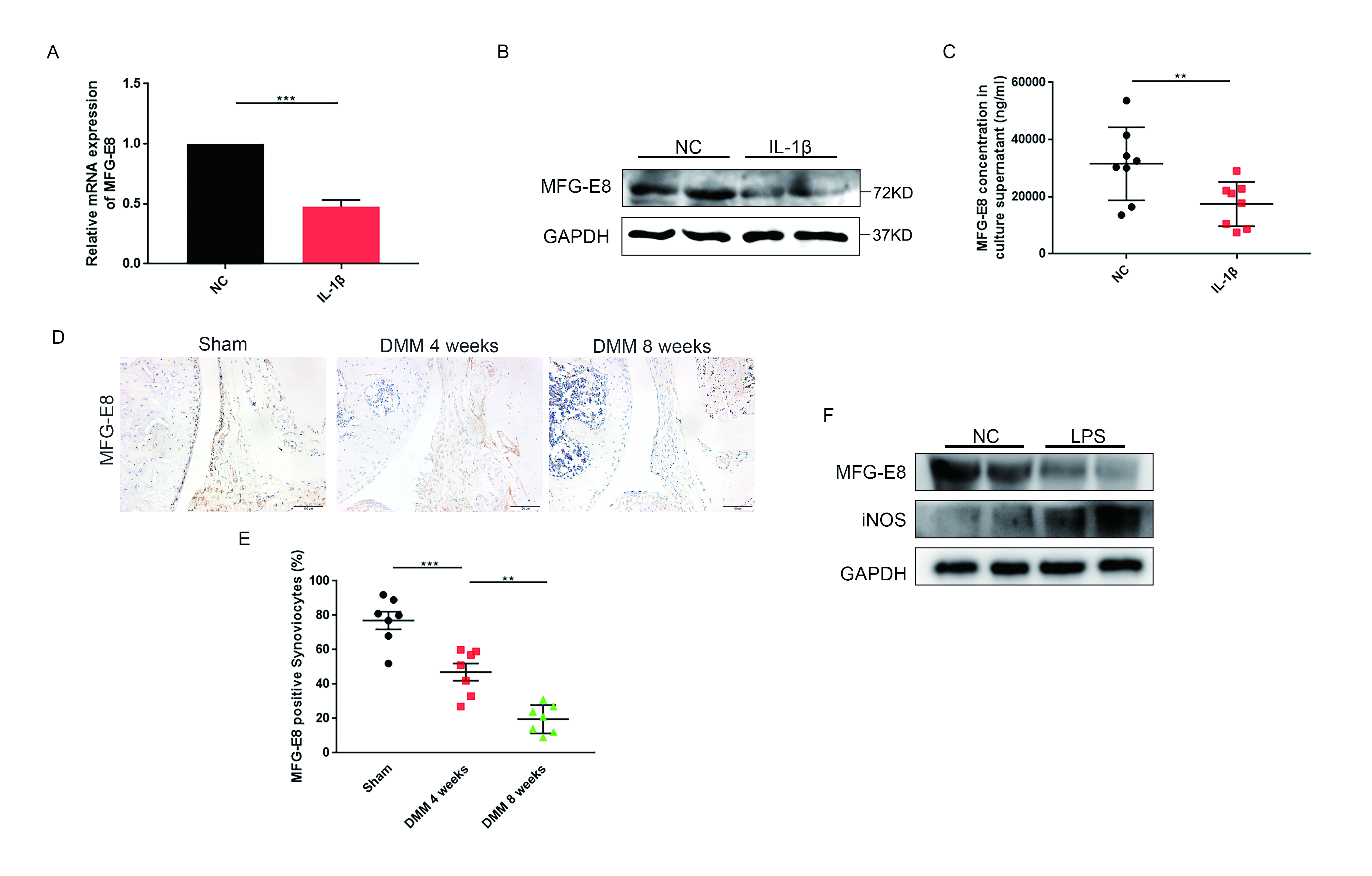

Supplement: Supplementary file 2 — Figure S1 [file 41419_2021_3800_MOESM2_ESM.tif]

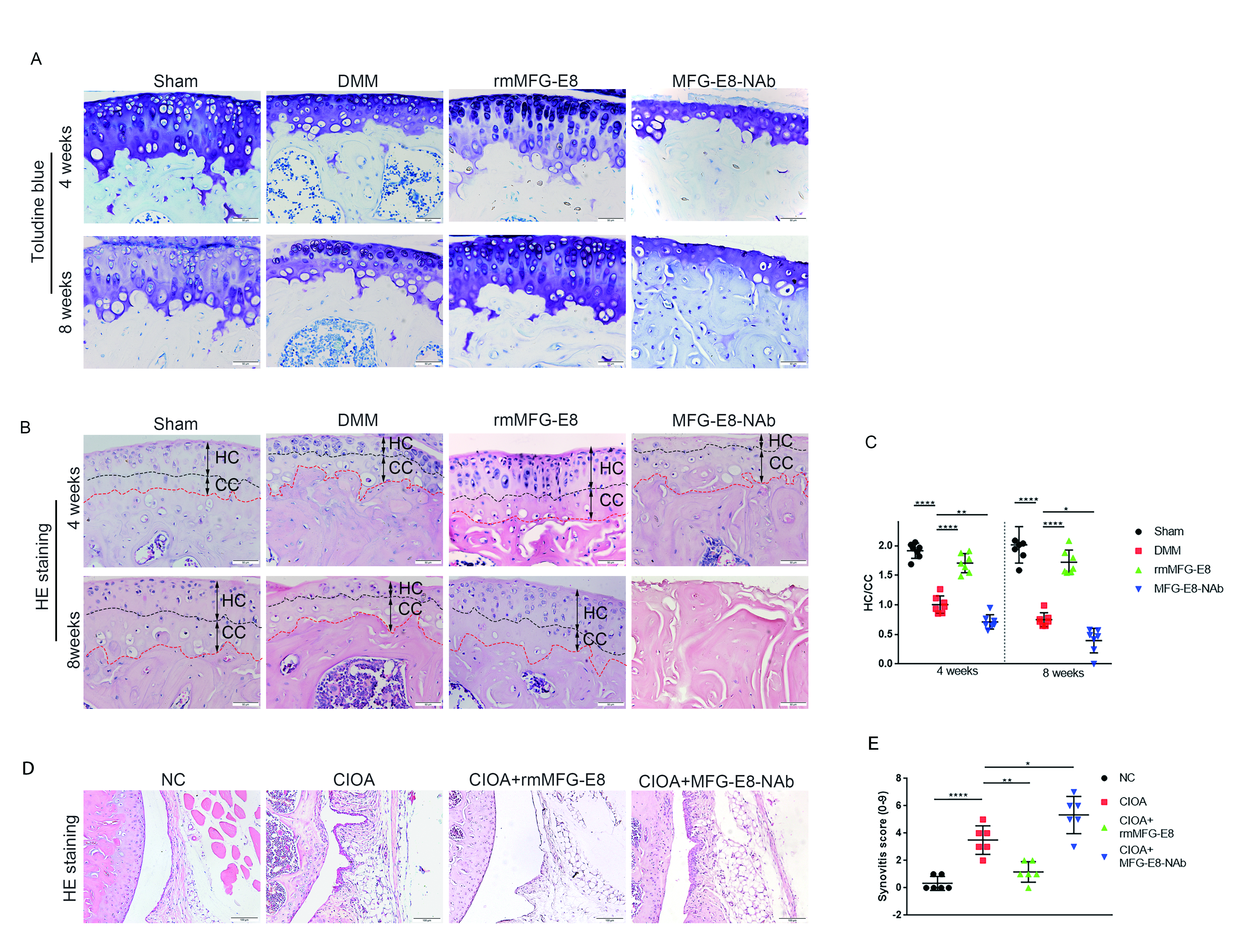

Supplement: Supplementary file 3 — Figure S2 [file 41419_2021_3800_MOESM3_ESM.tif]

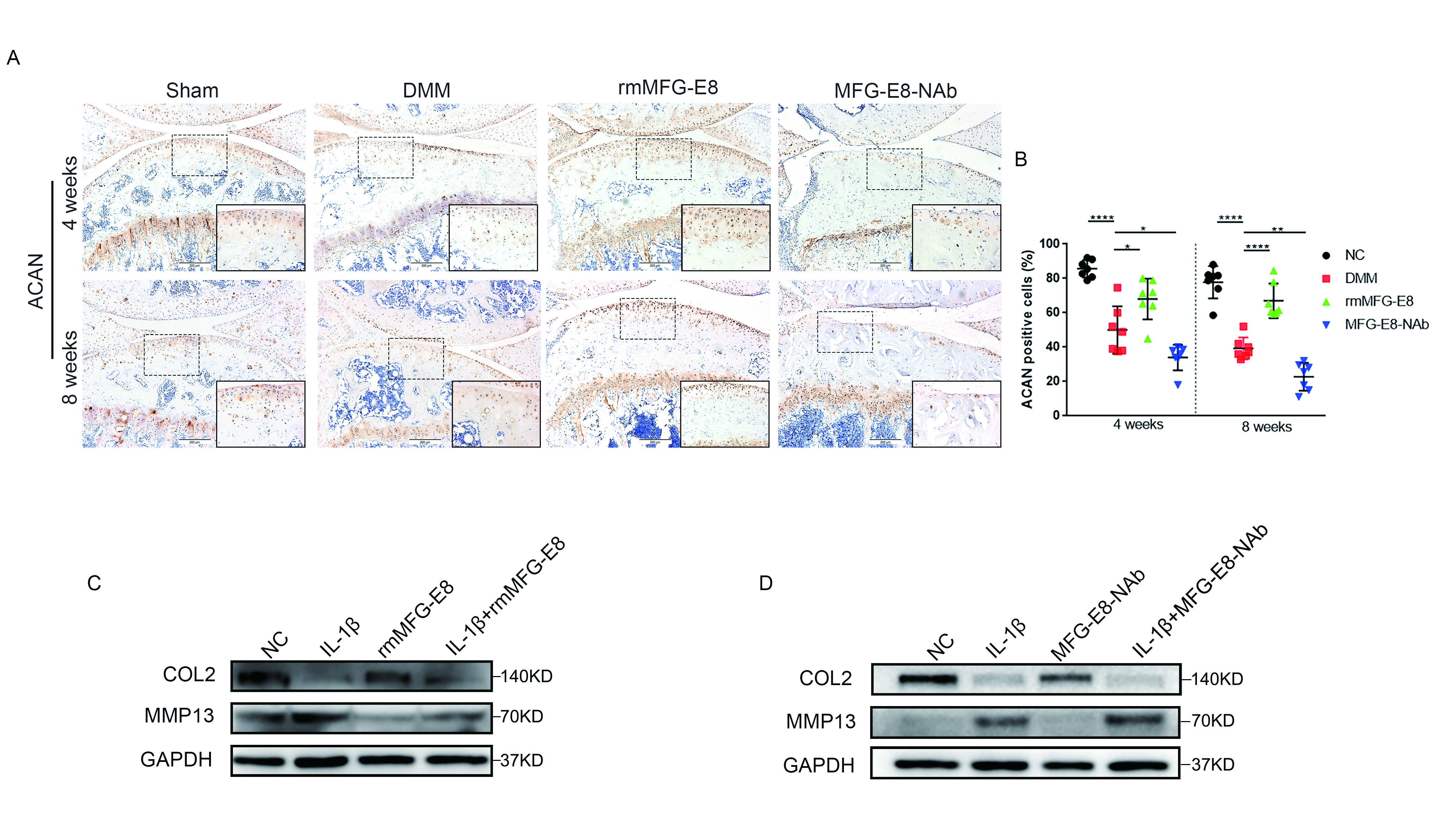

Supplement: Supplementary file 4 — Figure S3 [file 41419_2021_3800_MOESM4_ESM.tif]

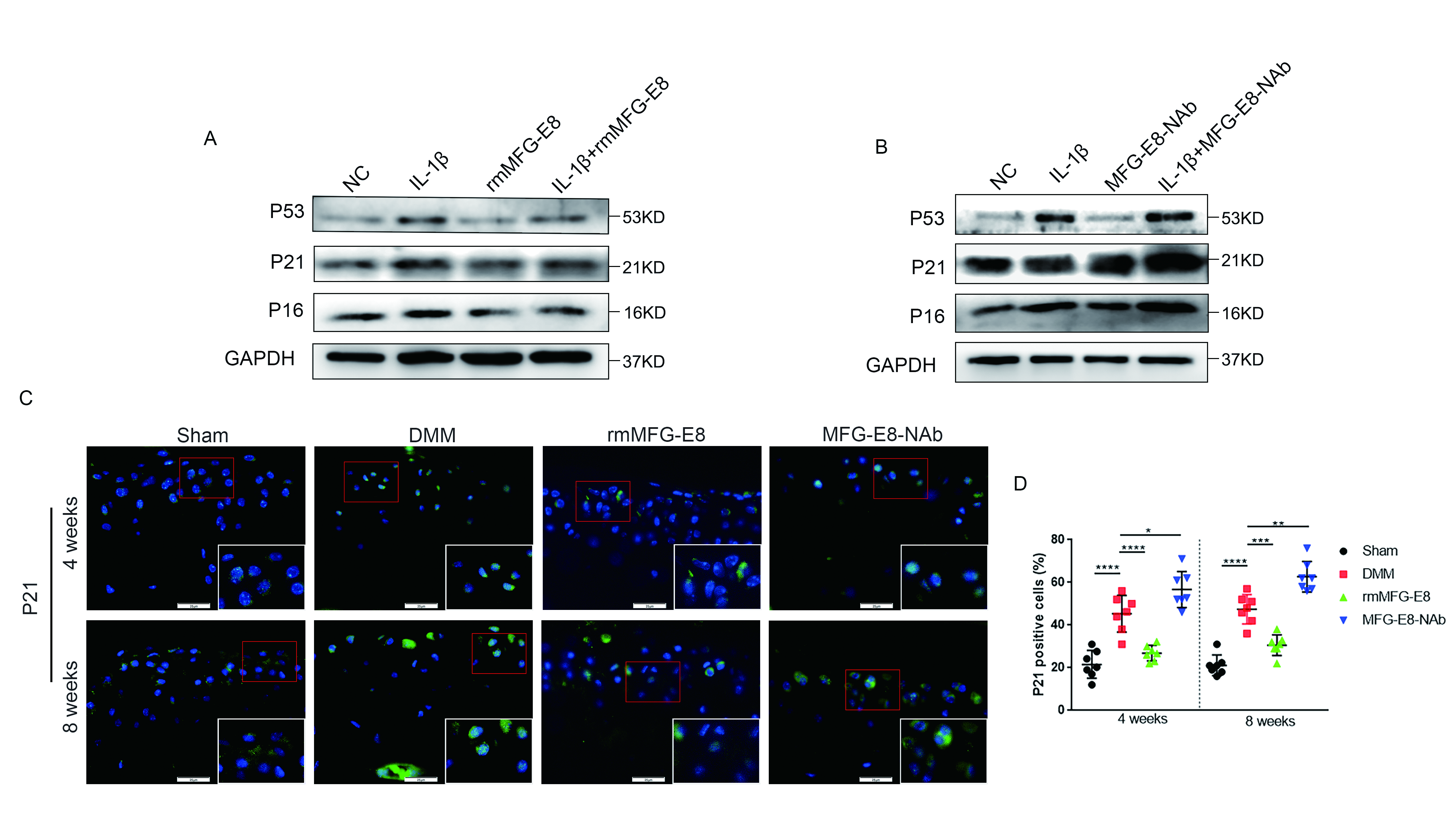

Supplement: Supplementary file 5 — Figure S4 [file 41419_2021_3800_MOESM5_ESM.tif]

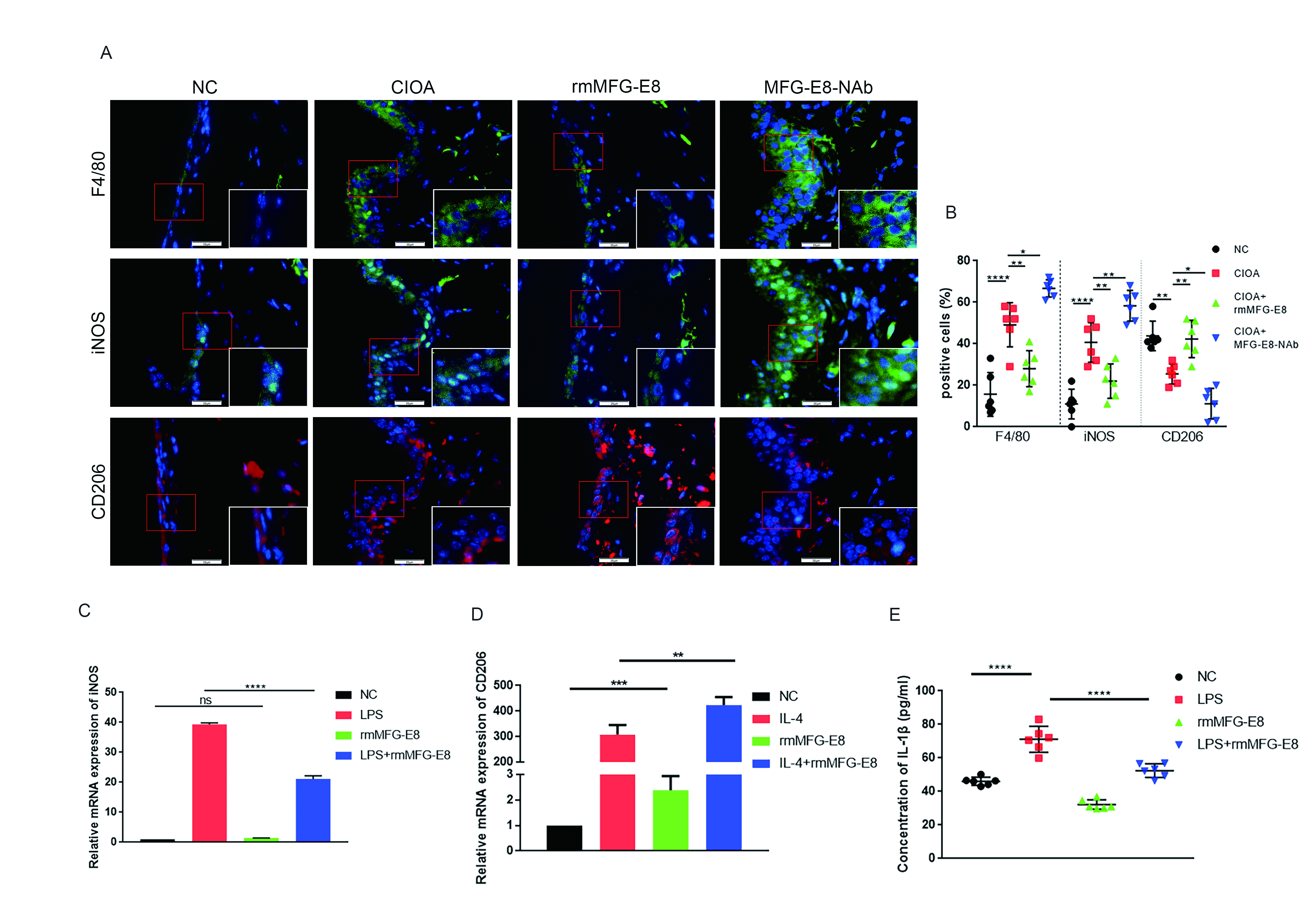

Supplement: Supplementary file 6 — Figure S5 [file 41419_2021_3800_MOESM6_ESM.tif]

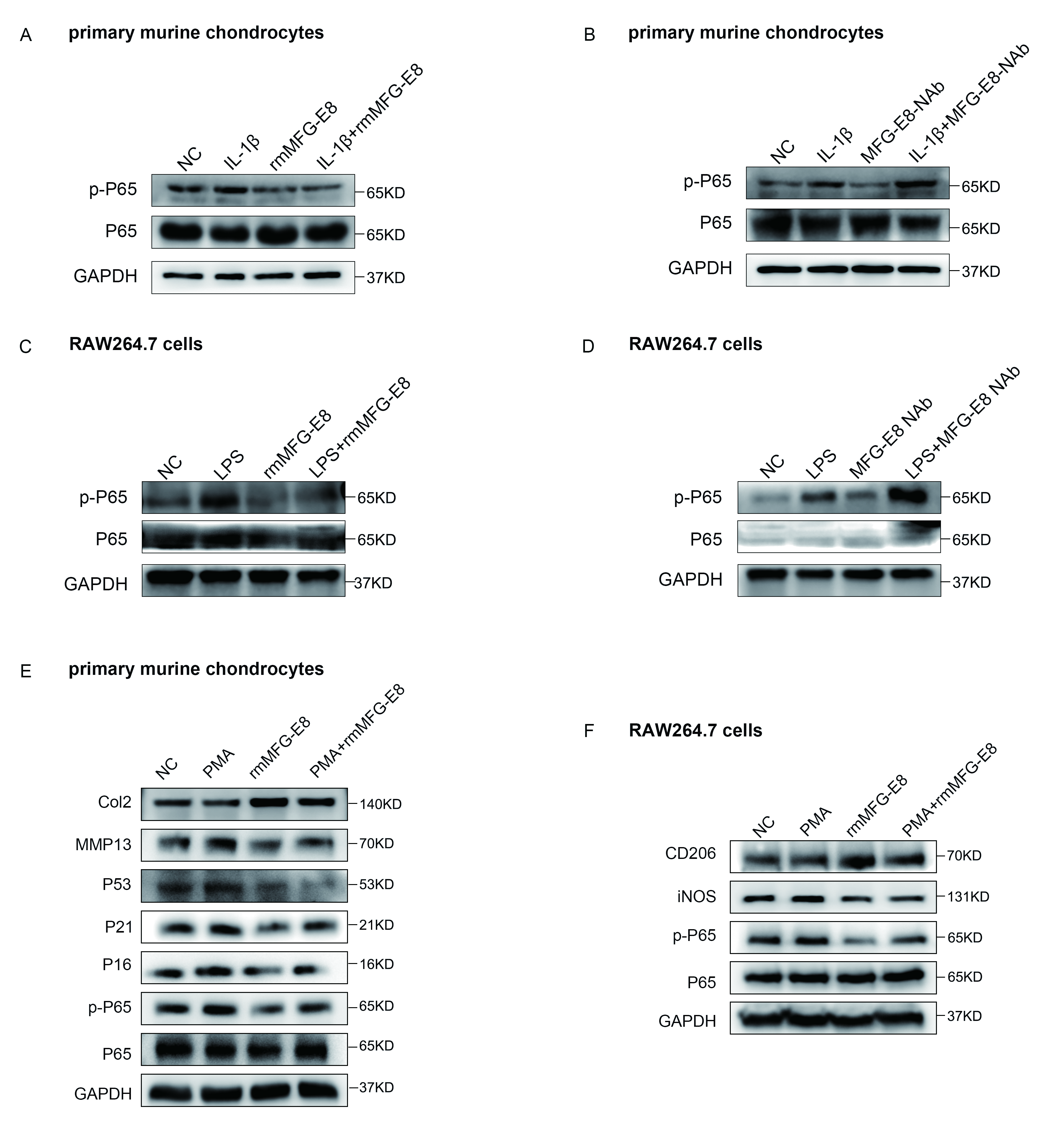

Supplement: Supplementary file 7 — Figure S6 [file 41419_2021_3800_MOESM7_ESM.tif]

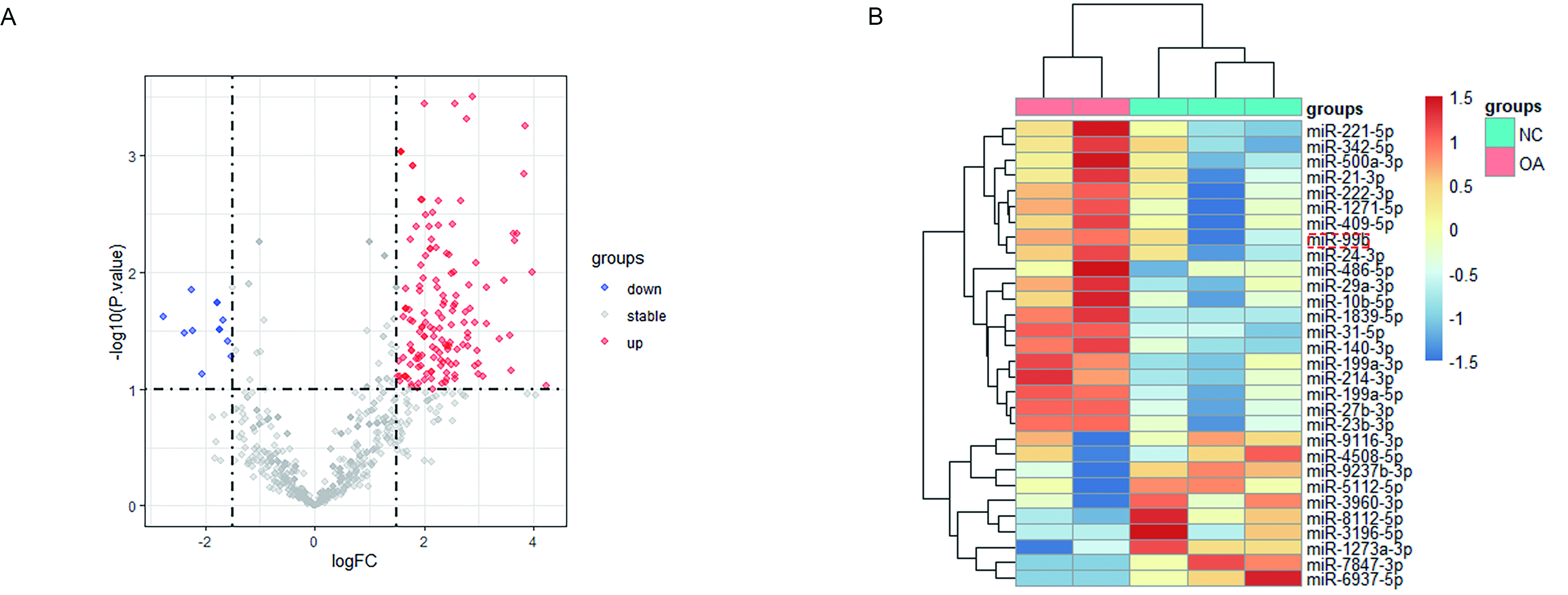

Supplement: Supplementary file 8 — Figure S7 [file 41419_2021_3800_MOESM8_ESM.tif]
